# Plasma metabolites mediate the effect of HbA1c on incident cardiovascular disease

**DOI:** 10.1002/clc.23243

**Published:** 2019-07-30

**Authors:** Xuesi Dong, Wei Zhou, Hu Li, Yuanming Fan, Xiaojian Yin, Yong Li, Feng Chen, Gaoxiang Ma

**Affiliations:** ^1^ Clinical Metabolomics Center, China Pharmaceutical University Nanjing China; ^2^ Department of Biostatistics School of Public Health, Nanjing Medical University Nanjing China; ^3^ State Key Laboratory of Natural Medicines School of Traditional Chinese Pharmacy, China Pharmaceutical University Nanjing China; ^4^ Department of Cardiology Nanjing University School Affiliated Nanjing Drum Tower Hospital Nanjing China; ^5^ Department of Cardiology the Affiliated Wujin Hospital of Jiangsu University Changzhou China

**Keywords:** cardiovascular disease, HbA1c, mediation analysis, metabolomics

## Abstract

**Background:**

We aim to discover whether HbA1c affects incident cardiovascular disease (CVD) through regulating endogenous metabolites.

**Methods and Results:**

Totally, 2019 plasma samples were analyzed by liquid chromatography‐quadrupole time‐of‐flight mass spectrometry. Logistic regression and linear regression were used to screen metabolites which were associated with both CVD and HbA1c. The VanderWeele's mediation approach was performed to assess the direct effect and indirect effect (IE) in the counterfactual model. Forty‐eight metabolites showed an association with both HbA1c and CVD risk. Forty‐four of the 48 metabolites worked as mediators mediated in HbA1c's effect on CVD (odds ratio [OR]_IE_ from 0.997 to 6.098, false discovery rate *q* < 0.05, mediated proportion from 0.4% to 85.4%). Pathway enrichment analysis indicated that different metabolic pathway showed significant IE (butanoate metabolism OR_IE_ = 1.058, mediated proportion = 16.0%; alanine, aspartate and glutamate metabolism OR_IE_ = 1.082, mediated proportion = 21.8%; TCA (citric acid) cycle metabolism OR_IE_ = 1.048, mediated proportion = 13.8%; phenylalanine metabolism OR_IE_ = 1.067, mediated proportion = 18.4%; glycerophospholipid metabolism OR_IE_ = 3.007, mediated proportion = 82.2%; all the *P* < .01).

**Conclusions:**

Our findings suggest that metabolites mediate the effect of HbA1c on incident CVD and provide a new study sight into pathogenesis of CVD.

## 1 INTRODUCTION

Cardiovascular disease (CVD) remains a leading cause of death worldwide, hence an exploration of CVD mechanism has become a top public health priority.^1^ Many researchers have confirmed the association between diabetes and CVD,^2^, ^3^, ^4^ and intensive glycemic control becomes the standard of care with diabetes complications.^5^, ^6^, ^7^ Previous studies have identified that the disorders of glucose and lipid metabolism are the crucial cause of CVD, and the abnormalities of glucose is the trigger.^8^, ^9^

As a major CVD risk factor, high level of HbA1c has been shown to activate the protein kinase C pathway,^10^ inducible nitric oxide synthase,^11^ and further induce inflammatory factors.^12^, ^13^ Glucose levels have a significant negative effect on endothelial function, and endothelial dysfunction is a key management of diabetes complications and CVD.^14^, ^15^ It is also informed that endothelial dysfunction can make a metabolic dysregulation.^16^ The metabolites represent one of the downstream end products of the environment's interaction with the genome‐transcriptome‐proteome, and the endogenous metabolites level can sensitively reflect phenotype fluctuation for CVD.^17^

However, the potential relationship among HbA1c, metabolites, and CVD is still unknown. Here, we carried a hospital‐based case control study with 2019 participants and aimed to explore a potential mechanism whether endogenous metabolites are involved in effect of HbA1c on incident CVD.

## 2 METHODS

### 2.1 Study population

A total of 2019 participants were recruited from the Affiliated Wujin Hospital of Jiangsu University during August 2009 and December 2015. Each participant underwent coronary angiography for confirmation the presence of CVD.

Plasma samples were collected before the coronary angiography surgery and quickly stored at −80°C for further metabolomic analyses. Blood biochemical indices were measured, and the history of diseases and smoking history were recorded using questionnaire. Patients with other cardiac‐related diseases, blood‐related disorders, infectious diseases, and malignant tumors were excluded. All subjects signed the informed consent forms. This study was approved by the ethics committee of Affiliated Wujin Hospital of Jiangsu University and complied with the Helsinki Declaration.

### 2.2 Sample preparation

To eliminate the protein in the plasma, 150 μL of acetonitrile was added to a 50 μL aliquot of plasma and vortexed for 10 seconds. Precipitated protein was subsequently removed by centrifugation (13 000 rpm, 10 minutes) at 4°C. Then, 150 μL of the supernatant was transferred to a tube and dried under a gentle stream of nitrogen gas at room temperature. Finally, the supernatant was reconstituted in 100 μL of aqueous acetonitrile (8:2, v/v) for LC/MS (Liquid Chromatography‐Mass Spectrometry) detection.

### 2.3 Quality control sample

To ensure data quality for metabolic profiling, quality control (QC) sample was proceeded. The detail process of QC referred to Fan et al.^18^

### 2.4 Metabolomics study

Liquid chromatographic separation was conducted using a 1290 Infinity System (Agilent Technologies, USA), with 100 × 2.1‐mm Zorbax Eclipse Plus 1.8‐mm C18 column maintained at 45°C. The mobile phase consisted of water with 5 mM ammonium acetate (A) and 10% aqueous acetonitrile with 5 mM ammonium acetate (B). Gradient program of elution was: 5 to 80% B at 0 to 7 minutes, 80 to 100% B at 7 to 12 minutes, 100% B at 12 to 13 minutes, and then back to initial conditions, and 2 minutes for equilibration. The sample volume injected was 1 μL and the flow rate was 0.4 mL/min.

The mass spectrometric detection was performed on an Agilent 6530 Q/TOF‐MS system (Agilent Technologies, USA) in positive mode. The parameters were set as: the fragmental voltage at 100 V, nebulizer gas at 35 psig, capillary voltage at 3500 V, drying gas flow rate at 10 L/minute, and temperature at 300°C. Reference masses at m/z 121.0509 and 922.0098 were introduced for accurate mass calibration.

MassHunter Workstation Software (version B.06.00; Agilent Technologies) was used to convert mass spectrometry data (.d) into data format (.mzdata) files. XCMS package (Scripps Center for Metabolomics and Mass Spectrometry, La Jolla, California) was used to conduct the data pre‐treatment, including nonlinear retention time alignment, peak discrimination, filtering, alignment, matching, and identification. The detailed information of the experiment has been described in previous study.^18^

### 2.5 Statistical process

#### 2.5.1 Data process

All metabolites concentrations were first log‐transformed prior to analyses to obtain approximately normal distributions. The false discovery rate (FDR) using Benjamini and Hochberg method was calculated to correct the multiple test adjustment. All tests were two‐sided, and *P* ≤ .05 were considered statistically significant unless stated otherwise.

#### 2.5.2 Metabolite‐based association analysis

Initially, we performed non‐conditional logistic regression to explore the association between HbA1c and CVD risk with adjusting or no‐adjusting potential covariates (age, gender, smoke and drink history). Then, the associations between (a) HbA1c and metabolites, (b) metabolites and CVD risk were assessed by multiple linear regression and non‐conditional logistic respectively, and the beta value (*β*), odds ratio (OR) and 95% confidence interval (95% CI) were calculated.

#### 2.5.3 Mediation analysis

In order to explore the potential causal mechanisms in “HbA1c – metabolites – CVD”, we further performed causal mediation analysis. Causal mediation analysis using VanderWeele's mediation model was applied to evaluate the indirect effect (IE) of HbA1c on CVD risk that was mediated through metabolites (explained by the change of metabolites per 1 SD increase of log‐transformed level), as well as the proportion of the effect mediated.^19^ The sum of the indirect and direct effects (DEs) is the total effect of HbA1c on CVD risk.

(1) $$\begin{array}{l} \text{logit}\left(P\right)=\theta_{0}+\theta_{\mathrm{HbA}1c}\times \mathrm{HbA}1c+\theta_{\text{Metabolite}}\times \text{Metabolite}\\ \;+\theta_{\text{covariates}}\times \text{Covariates}+e \end{array}$$

in this section, the *θ*_HbA1c_ represents the coefficient of HbA1c conditioned on covariates and metabolite, which also be regard as DE. The *θ*_Metabolite_ and *θ*_covariates_ represent the coefficients of metabolite and covariates, respectively. IE was calculated from formula (2):

(2) $$\mathrm{IE}=\theta_{\text{Metabolite}}\times \beta_{\mathrm{HbA}1c}$$

where *β*_HbA1c_was estimated by formula (3):

(3) $$\text{Metabolite}=\beta_{0}+\beta_{\mathrm{HbA}1c}\times \mathrm{HbA}1c+\beta_{\text{Covariates}}\times \text{Covariates}+e,\text{Weights}=w$$

In this section, *w*represents the weights, which were calculated for case and control respectively:

(4) $$w=\left\{\begin{array}{c} \text{prevalence}/r\;,\text{for case}\\ \left(1-\text{prevalence}\right)/\left(1-r\right)\;,\text{for control} \end{array}\right.$$

in which prevalence represents the prevalence of CVD among the at‐risk population, and *r* represents the proportion of CVD cases in our dataset. Monte Carlo bootstrapping with 1000 times was used to generate the standard errors.

#### 2.5.4 Pathway analysis

We classified the metabolites into different pathways using “*MetaboAnalyst*” (http://www.metaboanalyst.ca/), so that, an elaboration of comprehensive metabolites will be accomplished. Principal component analysis was used to extract the first principal component (PC1) from metabolites by pathways. Afterwards, mediation analysis was conducted to seek for the potential relationship among HbA1c, metabolic pathways, and CVD.

#### 2.5.5 Stratified analysis

The whole participants were divided into young (age < 65) and old (≥65) groups by age, we conducted the mediation analyses in subsets. Potential confounders incorporating age, gender, smoking and drinking history were used as covariates and considerably adjusted in all models. All analyses and visualizations were conducted using R version 3.4.1 (The R Foundation).

## 3 RESULTS

### 3.1 Characteristics of sample

A total of 2019 subjects (1784 CVD patients and 235 at‐risk controls) were included in this study. Each sample underwent coronary angiography and plasma samples were analyzed by liquid chromatography‐quadrupole time‐of‐flight mass spectrometry. The mean age of 2019 participants was 62.9 ± 9.6 years, of which males and females were 1276 (63.1%) and 743 (36.9%), 75% was non‐smoker, and 93% was non‐drinker. No significant differences were observed in smoke and drink history distributions among CVD and non‐CVD groups. Age and gender were found as unbalanced distributions between CVD and non‐CVD subjects (Figure S1).

### 3.2 Metabolite‐based association analysis

**Figure 1:**
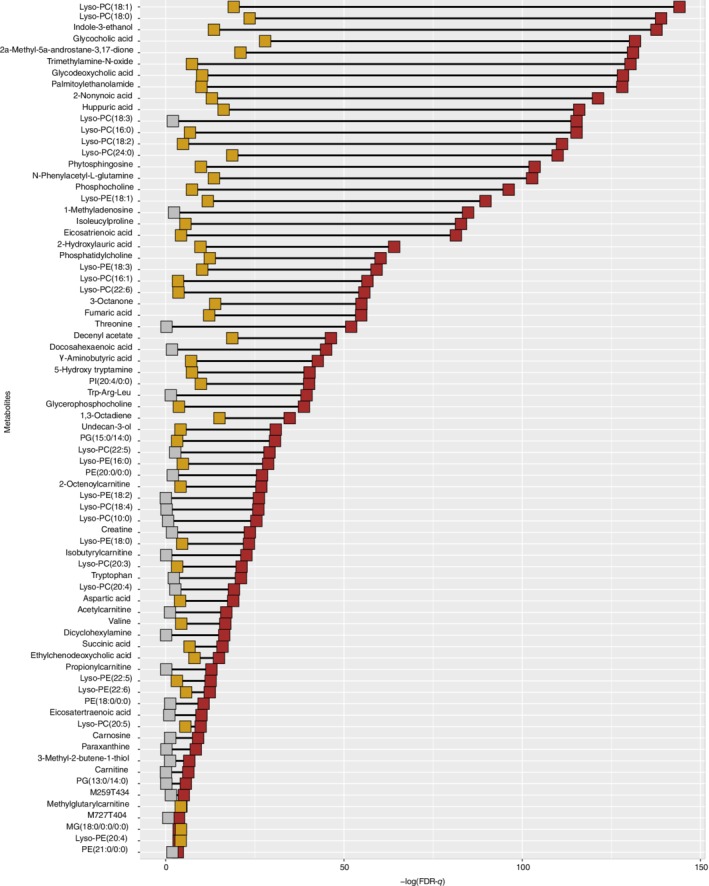
Metabolites associated with incident cardiovascular disease (CVD) (red blocks) and HbA1c (yellow blocks). All metabolites were nominally associated with incident CVD. Among those metabolites, gray blocks represented no significant association with HbA1c. FDR‐*q* values were calculated, and 0.05 was threshold. *means that the metabolites were tentatively identified with reference compounds

The risk of CVD was increased with the raising level of HbA1c (OR = 1.79, 95% CI = [1.48, 2.18], *P* = 3.79 × 10^−9^), which was consistent with a previous follow‐up study.^20^ This positive association was further validated when the model was further adjusted for covariates (OR = 1.86, 95% CI = [1.54, 2.32], *P* = 2.91 × 10^−9^). A total of 2059 positive ions were confirmed in our study. We performed a non‐condition logistic regression to uncover the relationship between CVD risk and metabolism features, and identified 176 metabolites associated with the incident CVD (*P* ≤ 0.05). Seventy‐five metabolites reached statistical significance after correction for multiple testing by FDR‐*q* ≤ 0.05. Acting as CVD risk factors, those metabolites might be influenced by HbA1c. We further explored the relationship between each significant metabolite and HbA1c. Out of 75 metabolites, 48 were then detected to be affected by HbA1c (*β* from −10.18 to 3.34, FDR‐*q* ≤ 0.05) (Table S1, Figure 1).

### 3.3 Casual inference analysis

Given these association between HbA1c, metabolites, and CVD risk, it may be possible that metabolites act as mediators of the effect of HbA1c on CVD risk (Figure S2). To verify the hypothesis, we performed a causal inference test using VanderWeele's mediation model and bootstrap methods. As shown in Table 1, among those 48 metabolites which were significantly associated with both CVD and HbA1c, 44 metabolic ions were found mediating the effect of HbA1c initiating CVD risk, with a significant IE (FDR‐*q* ≤ 0.05). In detail, HbA1c decreased the level of methylglutarylcarnitine which further reduced CVD risk. In another word, methylglutarylcarnitine alleviated the effect of HbA1c on CVD risk (masking effect), and the mediated proportion was not estimated due to the opposite signs (OR_IE_ = 0.997, 95% CI = [0.999, 0.994], FDR‐*q* = 0.026). 2α‐Methyl‐5α‐androstane‐3,17‐dione and glycocholic acid revealed a wholly mediate effect (2α‐Methyl‐5α‐androstane‐3,17‐dione: OR_IE_ = 1.023, 95% CI = [1.017, 1.029], FDR*‐q* = 9.24 × 10^−13^; Glycocholic acid: OR_IE_ = 1.246, 95% CI = [1.183, 1.320], FDR‐*q* = 1.07 × 10^−13^). More generally, adjusting with 2α‐Methyl‐5α‐androstane‐3,17‐dione and glycocholic acid, the effect of HbA1c on CVD was non‐significantly. Besides, the remaining 41 metabolites showed a positive IE explaining partial phenotypic variance (OR_IE_ = 1.001 to 6.098, FDR‐*q* ≤ 0.05, mediated proportion = 0.4% to 85.4%).

**Table 1:** HbA1c associated with incident cardiovascular disease mediated by metabolites

Metabolites	Indirect effect (IE)	FDR‐*q*_IE_	Mediated proportion (%)
1,3‐Octadiene	1.050 (1.031, 1.074)	8.38 × 10^−6^	15.2
2‐Hydroxylauric acid	1.009 (1.005, 1.014)	2.02 × 10^−4^	3.2
2‐Nonynoic acid	1.012 (1.007, 1.018)	6.08 × 10^−5^	4.4
2‐Octenoylcarnitine	1.001 (0.999, 1.002)	5.31 × 10^−2^	a
2α‐Methyl‐5α‐androstane‐3,17‐dione	1.023 (1.017, 1.029)	9.24 × 10^−13^	b
3‐Octanone	1.038 (1.023, 1.052)	1.33 × 10^−6^	11.9
5‐Hydroxy tryptamine	1.002 (1.001, 1.004)	1.31 × 10^−2^	0.8
*Aspartic acid	1.040 (1.007, 1.087)	4.93 × 10^−2^	11.7
Decenyl acetate	1.002 (1.001, 1.003)	2.02 × 10^−4^	0.7
*Eicosatrienoic acid	1.008 (1.003, 1.015)	1.38 × 10^−2^	3
*Ethylchenodeoxycholic acid	1.021 (1.008, 1.036)	6.21 × 10^−3^	6.5
*Fumaric acid	1.011 (1.006, 1.015)	4.90 × 10^−5^	3.3
*Glycerophosphocholine	1.024 (1.003, 1.047)	3.48 × 10^−2^	6.9
*Glycocholic acid	1.246 (1.183, 1.320)	1.07 × 10^−13^	b
*Glycodeoxycholic acid	1.007 (1.004, 1.010)	1.92 × 10^−5^	3
*Hippuric acid	1.012 (1.007, 1.014)	1.71 × 10^−7^	3.7
Indole‐3‐ethanol	1.001 (1.001, 1.001)	6.85 × 10^−7^	0.4
*Isoleucylproline	1.004 (1.002, 1.007)	3.12 × 10^−3^	1.5
*LysoPC(16:0)	6.098 (2.201, 17.253)	1.78 × 10^−3^	85.4
*LysoPC(16:1)	1.024 (1.003, 1.046)	3.21 × 10^−2^	7.5
*LysoPC(18:0)	2.527 (1.956, 3.323)	8.48 × 10^−11^	79.6
*LysoPC(18:1)	2.942 (2.032, 4.255)	1.00 × 10^−7^	82.2
*LysoPC(18:2)	1.677 (1.182, 2.373)	6.32 × 10^−3^	61.2
*LysoPC(20:3)	1.025 (1.002, 1.052)	4.91 × 10^−2^	7.6
LysoPC(20:5)	1.022 (1.009, 1.045)	2.01 × 10^−2^	6.9
LysoPC(22:6)	1.034 (1.004, 1.064)	3.12 × 10^−2^	9.9
LysoPC(24:0)	1.008 (1.005, 1.011)	1.18 × 10^−6^	3.2
*LysoPE(16:0)	1.022 (1.006, 1.044)	1.35 × 10^−2^	6.7
*LysoPE(18:0)	1.006 (1.002, 1.012)	2.60 × 10^−2^	2.1
*LysoPE(18:1)	1.011 (1.006, 1.016)	5.55 × 10^−5^	4.1
LysoPE(18:3)	1.006 (1.003, 1.009)	2.02 × 10^−4^	2
LysoPE(20:4)	1.015 (1.002, 1.031)	4.83 × 10^−2^	4.7
LysoPE(22:5)	1.001 (0.999, 1.003)	1.95 × 10^−1^	a
LysoPE(22:6)	1.008 (1.002, 1.016)	3.22 × 10^−2^	2.8
Methylglutarylcarnitine	0.997 (0.999, 0.994)	2.63 × 10^−2^	c
MG(18:0/0:0/0:0)	1.006 (1.001, 1.014)	7.36 × 10^−2^	a
N‐Phenylacetyl‐L‐glutamine	1.231 (1.133, 1.349)	1.22 × 10^−5^	42.7
*Palmitoylethanolamide	1.006 (1.003, 1.009)	2.02 × 10^−4^	2.7
PG(15:0/14:0)	1.014 (1.002, 1.029)	4.81 × 10^−2^	4.4
Phosphatidylcholine	1.006 (1.004, 1.009)	1.22 × 10^−5^	2.2
*Phytosphingosine	1.112 (1.059, 1.172)	1.39 × 10^−4^	29.3
Phosphocholine	1.045 (1.021, 1.074)	1.32 × 10^−3^	12.5
PI(20:4/0:0)	1.033 (1.017, 1.049)	1.47 × 10^−4^	9.7
Succinic acid	1.047 (1.018, 1.087)	9.32 × 10^−3^	13.6
Trimethylamine‐N‐oxide	1.014 (1.006, 1.022)	1.75 × 10^−3^	5.3
Undecan‐3‐ol	1.007 (0.999, 1.022)	2.12 × 10^−1^	a
*Valine	1.384 (1.069, 1.883)	3.25 × 10^−2^	53
*γ‐Aminobutyric acid	1.021 (1.009, 1.035)	2.32 × 10^−3^	6.6

Abbreviation: FDR, false discovery rate.

*Means that the metabolites were tentatively identified with reference compounds.

a Mediated proportion cannot be estimated because the IE is not significant.

b Mediated proportion cannot be estimated because the DE is not significant.

c Mediated proportion cannot be estimated because the opposite signs with DE and IE.

### 3.4 Pathway analysis

**Figure 2:**
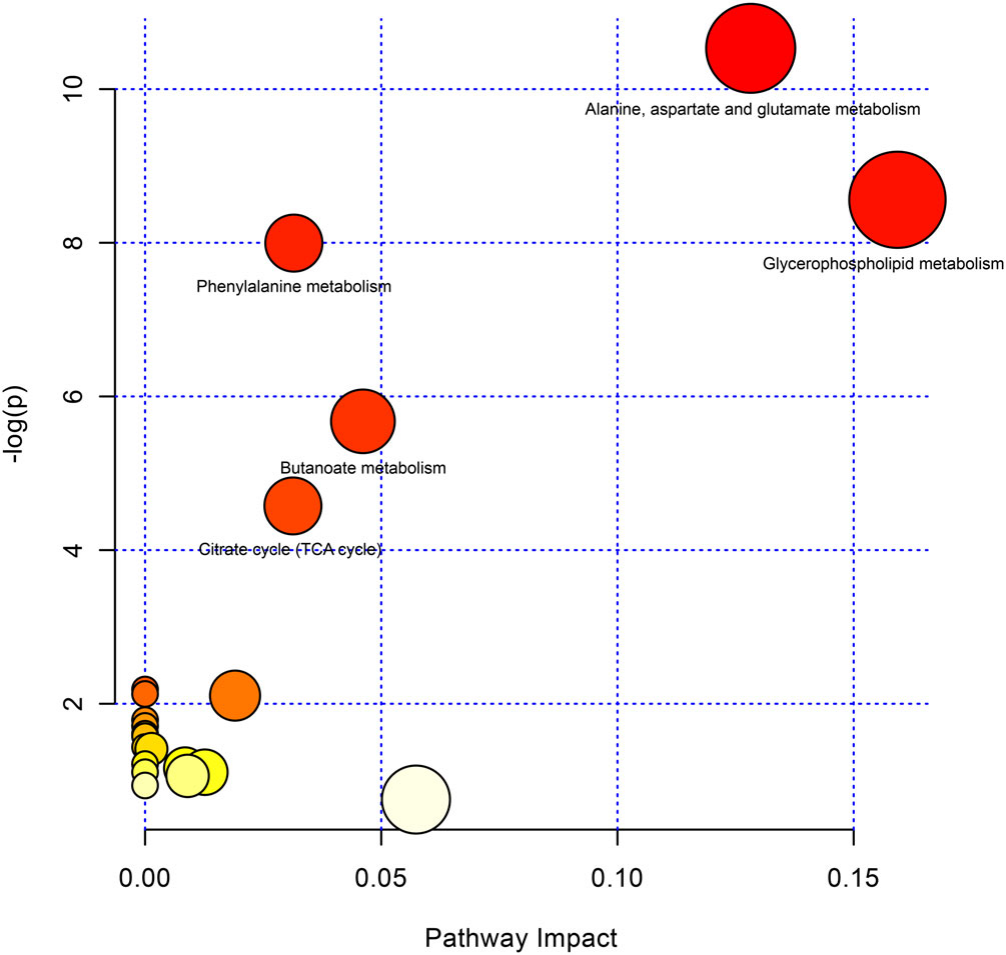
Metabolic pathway analysis was performed to enrich significant metabolites into different pathways

Considering an overall interpretation for metabolic IDE, pathway enrichment was investigated. Figure 2 showed all enriched metabolic pathways according to *P* values, and we extracted PC1s from the corresponding five metabolic pathways (*Alanine*, *aspartate and glutamate metabolism*, *glycerophospholipid metabolism*, *phenylalanine metabolism*, *butanoate metabolism*, and *citrate cycle metabolism*). The corresponding PC1s' proportion of variance was 0.950, 0.898, 0.994, 0.897, and 0.947, respectively, which indicated an appropriate representativeness of pathway metabolites. We assessed the proportion of HbA1c effect on risk of CVD that was mediated through metabolic PC1s. As shown in Table 2, Butanoate metabolism OR_IE_ = 1.058, FDR‐*q* = 0.001, mediated proportion = 16%; alanine, aspartate and glutamate metabolism OR_IE_ = 1.082, FDR‐*q* = 0.002, mediated proportion = 21.8%; TCA cycle metabolism OR_IE_ = 1.048, FDR‐*q* = 0.003, mediated proportion = 13.8%; phenylalanine metabolism OR_IE_ = 1.067, FDR‐*q* = 0.004, mediated proportion = 18.4%; glycerophospholipid metabolism OR_IE_ = 3.007, FDR‐*q* = 5.72 × 10^−9^, mediated proportion = 82.2%. The IE among those pathways ranged from 1.048 to 3.001, which averagely explained 30.4% total effect (IE + DE). The linear combination of metabolites in certain pathway uncovered a potential mediator in HbA1c caused CVD.

**Table 2:** HbA1c associated with incident cardiovascular disease mediated by first principal component in metabolic pathways

Metabolites	Pathway	PC1 (%)	Indirect effect (IE)	*P*_IE_	Mediated proportion (%)
*Fumaric acid	Butanoate metabolism	95.0	1.058 (1.022, 1.095)	1.47 × 10^−3^	16.0
*γ‐Aminobutyric acid					
*Succinic acid					
*Aspartic acid	Alanine, aspartate and glutamate metabolism	89.8	1.082 (1.035, 1.146)	2.39 × 10^−3^	21.8
*γ‐Aminobutyric acid					
*Fumaric acid					
*Succinic acid					
*Fumaric acid	Citrate cycle (TCA cycle)	99.4	1.048 (1.022, 1.087)	2.52 × 10^−3^	13.8
*Succinic acid					
*Hippuric acid	Phenylalanine metabolism	89.7	1.067 (1.035, 1.116)	3.87 × 10^−3^	18.4
N‐Phenylacetyl‐L‐glutamine					
*Fumaric acid					
*Succinic acid					
Phosphatidylcholine	Glycerophospholipid metabolism	94.7	3.007 (2.079, 4.362)	5.72 × 10^−9^	82.2
*LysoPC(18:1)					
Phosphocholine					
Glycerophosphocholine					

*Means that the metabolites were tentatively identified with reference compounds.

### 3.5 Stratified analysis

Based on the age division standard from world health organization (WHO), we divided participants into young (<65) and old (≥65) groups. Re‐extracting PC1s from both young and old groups, we obtained analogous results in 5 metabolic pathways (Tables S2 and S3). Compared with total participants, five metabolic pathways showed a consistent result in young group (butanoate metabolism OR_IE_ = 1.050, *P* = .01, mediated proportion = 14.7%; alanine, aspartate and glutamate metabolism OR_IE_ = 1.062, *P* = 0.025, mediated proportion = 17.9%; TCA cycle _IE_ = 1.049, *P* = 0.011, mediated proportion = 14.5%; phenylalanine metabolism OR_IE_ = 1.075，*P* = .003, mediated proportion = 20.6%; glycerophospholipid metabolism OR_IE_ = 3.561，*P* = 8.25 × 10^−8^, mediated proportion = 86.5%). However, butanoate metabolism, citrate cycle, and phenylalanine metabolism in old group failed (butanoate metabolism OR_IE_ = 1.040，*P* = .192; alanine, aspartate and glutamate metabolism OR_IE_ = 1.129，*P* = .023, mediated proportion = 29.2%; TCA cycle OR_IE_ = 1.039，*P* = .197; phenylalanine metabolism OR_IE_ = 1.051, *P* = .142; glycerophospholipid metabolism OR_IE_ = 2.557, *P* = .003, mediated proportion = 70.2%).

## 4 DISCUSSION

As previous reported, metabolic biomarkers always play a critical role in CVD.^20^ Hyperglycemia, as measured by HbA1c level, is confirmed as an independent risk factor for CVD, and leads to a poor prognosis. Owing to the unknown mechanism, we investigated relationship among CVD, extensive plasma metabolites and HbA1c. Overall, we consider that HbA1c might induce CVD via metabolites contributions. These metabolites may act as an important role in the causal pathway from HbA1c to CVD risk. To our knowledge, this is the first casual inference study involving “HbA1c‐Metabolites‐CVD risk”.

In this study, significant IEs were identified among 42 metabolites independent from established CVD risk factor, and nine metabolites belonged to lysophosphatidylcholine (LPC) category. Ganna et al^21^ discovered a strong negative association between LPC and incident CVD in independent studies, Stegemann et al^22^ confirmed that high levels of individual species of LPC were predictive factor for CVD over a 10‐year observation period. In additional, Sattler declared that the level of LPC tightly associated with type 2 diabetes that were independently confirmed in the European Prospective Investigation into Cancer and Nutrition Potsdam cohort.^23^ Consisting with the potentially relationship among HbA1c, LPC, and CVD, HbA1c in this study was also found to decrease the level of LPC relative ions, which indirectly caused elevation of CVD risk.

Pathway analysis classified metabolic mediators into five categories, and principal analysis assembles those metabolites by pathway. The blood glucose can be metabolized into small molecule metabolites and then enter the TCA‐cycle,^24^ which is directly coupled to myocardial oxygen consumption. This cycle plays a key role in cell metabolism and the levels of the involved metabolites can also be affected by other physiological factors (HbA1c).^25^ Recent study demonstrates that residents living in the pollution‐affected area always be with higher levels of urine metabolite profiles, incorporating phenylalanine metabolism and alanine, aspartate and glutamate metabolism. Those metabolites are linked to increased oxidative stress, including oxidative and nitrative DNA damage, and lipid peroxidation.^26^, ^27^ It was well known that high level glucose contributes to oxidative stress, which caused oxidative injury and dysfunction of the vascular endothelium as an indicator in many vascular diseases in early stage. DeRatt et al found that a high plasma cystathionine concentration in stable angina pectoris was associated with higher glucose, and phenylalanine concentrations due to greater catabolism.^28^ Furthermore, glycerophospholipid metabolism and butanoate metabolism are also relevant to oxidative stress, and glycerophospholipid metabolism has been confirmed to be associated with CVD risk.^29^, ^30^, ^31^

This research is absence in external validation. In order to enhance the reliability, we performed a bootstrap when estimating the SE of DE and IE, and a stratified analysis was performed to validate the results. During stratified analysis, we confirmed the robustness performance of our results in young group, but three metabolic pathways (*butanoate metabolism, TCA cycle, phenylalanine metabolism*) in old group failed to revel a significant IE.

In this study, we captured 48 metabolites associated with both HbA1c and CVD risk and tested their mediation effect. It was revealed that metabolite may act as a mediator between the connection of HbA1c and CVD incidence and has a potential regulatory mechanism. we also recognized some limitations here. First, because of the uncommercial reference compounds obtained, identification of metabolites is still a challengeable work. Second, future prospective confirmation in independent cohorts is warranted. Third, the targeted metabolomics was not done, and only the untargeted metabolomics for screening of significant metabolites was studied. Fourth, the distributions of age and gender between case and control subjects were identified, indeed we adjusted those potential confounders among all models.

## 5 CONCLUSIONS

Metabolites were the downstream end products in endogenous matrix. High level HbA1c contributed to incident CVD, which may be mediated by metabolite functions. Explanations of the relationship among HbA1c, metabolites and CVD were beneficial to the etiological study of CVD.

## CONFLICT OF INTEREST

The authors declare no potential conflict of interest.

## AUTHORS' CONTRIBUTIONS

F.C., Y.L., and G.M. conceived and designed the research study. X.D. and W.Z. analyzed the data and finished the manuscript writing. F.C. oversaw all parts of the analysis. X.Y. and Y.F. contributed to project development and manuscript revision. H.L. revised the manuscript All authors approved the final submission.

## Supporting information


**Figure S1.** Clinical characteristics.
**Figure S2**. Schematic diagram of mediation analysis.
**Table S1**. Metabolites associated with incident cardiovascular disease and HbA1c.
**Table S2**. HbA1c associated with incident cardiovascular disease mediated by first principal component in various metabolic pathway in young group.
**Table S3**. HbA1c associated with incident cardiovascular disease mediated by first principal component in various metabolic pathway in old group.
