# Supplementary material for: Plasma metabolites mediate the effect of HbA1c on incident cardiovascular disease
Source: Clin Cardiol. 2019 Jul 30;42(10):934–41. doi: 10.1002/clc.23243 (PMC6788485; doi:10.1002/clc.23243)

**Figure legends**

Figure S1. Clinical characteristics.

Figure S2. Schematic diagram of mediation analysis.

Table S1. Metabolites associated with incident cardiovascular disease and HbA1c

| Metabolites | Beta^a^ | FDR *q*^a^ | Beta^b^ | FDR *q*^b^ |
| --- | --- | --- | --- | --- |
| ***Lyso-PC(18:1)** | -0.31 (-0.35, -0.28) | 2.63×10^-63^ | -0.02 (-0.03, -0.01) | 5.24×10^-09^ |
| ***Lyso-PC(18:0)** | -0.15 (-0.17, -0.13) | 4.65×10^-61^ | -0.03 (-0.04, -0.02) | 6.02×10^-11^ |
| Indol×10-3-ethanol | -117.59 (-131.30, -103.87) | 1.67×10^-60^ | -10.18 (-13.98, -6.36) | 1.36×10^-06^ |
| ***Glycocholic acid** | -2.20 (-2.46, -1.94) | 6.89×10^-58^ | -0.17 (-0.22, -0.13) | 7.76×10^-13^ |
| 2α-Methyl-5α-androstan×10-3,17-dione | -23.27 (-26.05, -20.48) | 1.20×10^-57^ | -1.41 (-1.83, -1.00) | 7.91×10^-10^ |
| Trimethylamin×10-N-oxide | 21.01 (18.49, 23.54) | 2.46×10^-57^ | 0.73 (0.34, 1.11) | 6.34×10^-04^ |
| ***Glycodeoxycholic acid** | -19.27 (-21.60, -16.93) | 2.01×10^-56^ | -1.30 (-1.88, -0.73) | 3.60×10^-05^ |
| ***Palmitoylethanolamide** | -25.77 (-28.90, -22.65) | 2.44×10^-56^ | -1.48 (-2.14, -0.82) | 4.46×10^-05^ |
| 2-Nonynoic acid | -10.62 (-11.95, -9.30) | 2.21×10^-53^ | -0.88 (-1.22, -0.54) | 2.37×10^-06^ |
| ***Huppuric acid** | 87.57 (76.42, 98.73) | 4.30×10^-51^ | 3.34 (2.21, 4.47) | 8.96×10^-08^ |
| ***Lyso-PC(16:0)** | -0.05 (-0.06, -0.04) | 9.23×10^-51^ | -0.03 (-0.04, -0.01) | 1.12×10^-03^ |
| Lyso-PC(18:3) | -17.22 (-19.42, -15.01) | 9.23×10^-51^ | -0.51 (-1.12, 0.09) | 1.33×10^-01^ |
| ***Lyso-PC(18:2)** | -0.118 (-0.13, -0.10) | 5.37×10^-49^ | -0.01 (-0.02, -0.00) | 7.80×10^-03^ |
| Lyso-PC(24:0) | -20.65 (-23.36, -17.95) | 1.83×10^-48^ | -2.01 (-2.64, -1.38) | 7.81×10^-09^ |
| ***Phytosphingosine** | 1.06 (0.92, 1.21) | 1.17×10^-45^ | 0.08 (0.04, 0.11) | 5.17×10^-05^ |
| N-Phenylacetyl-L-glutamine | 1.42 (1.23, 1.62) | 2.37×10^-45^ | 0.08 (0.05, 0.11) | 1.36×10^-06^ |
| Phosphocholine | -0.95 (-1.09, -0.82) | 1.69×10^-42^ | -0.09 (-0.13, -0.04) | 6.34×10^-04^ |
| ***Lyso-PE(18:1)** | -4.85 (-5.55, -4.15) | 1.12×10^-39^ | -0.55 (-0.78, -0.33) | 7.61×10^-06^ |
| 1-Methyladenosine | 13.89 (11.82, 15.96) | 1.50×10^-37^ | 0.42 (-0.03, 0.87) | 9.89×10^-02^ |
| ***Isoleucylproline** | -13.17 (-15.16, -11.19) | 1.09×10^-36^ | -0.75 (-1.22, -0.29) | 4.01×10^-03^ |
| ***Eicosatrienoic acid** | -3.71 (-4.27, -3.14) | 4.58×10^-36^ | -0.23 (-0.40, -0.06) | 1.37×10^-02^ |
| 2-Hydroxylauric acid | 4.97 (4.12, 5.82) | 1.49×10^-28^ | 0.53 (0.29, 0.78) | 5.87×10^-05^ |
| Phosphatidylcholine | -5.96 (-7.01, -4.91) | 6.75×10^-27^ | -0.88 (-1.22, -0.53) | 4.31×10^-06^ |
| Lyso-PE(18:3) | -5.88 (-6.93, -4.84) | 2.10×10^-26^ | -0.77 (-1.11, -0.43) | 3.60×10^-05^ |
| ***Lyso-PC(16:1)** | -0.77 (-0.91, -0.63) | 2.71×10^-25^ | -0.05 (-0.09, -0.01) | 3.18×10^-02^ |
| Lyso-PC(22:6) | -0.49 (-0.58, -0.40) | 6.44×10^-25^ | -0.04 (-0.07, -0.01) | 2.81×10^-02^ |
| 3-Octanone | -0.77 (-0.92, -0.63) | 1.45×10^-24^ | -0.15 (-0.20, -0.09) | 9.47×10^-07^ |
| ***Fumaric acid** | -3.41 (-4.04, -2.78) | 1.54×10^-24^ | -0.53 (-0.74, -0.32) | 5.05×10^-06^ |
| ***Threonine** | -0.73 (-0.86, -0.59) | 2.57×10^-23^ | -0.01 (-0.05, 0.03) | 8.06×10^-01^ |
| Decenyl acetate | -14.81 (-17.77, -11.85) | 7.58×10^-21^ | -3.56 (-4.68, -2.44) | 7.81×10^-09^ |
| Docosahexaenoic acid | -0.93 (-1.12, -0.74) | 2.94×10^-20^ | -0.05 (-0.12, 0.02) | 1.76×10^-01^ |
| ***γ-Aminobutyric acid** | -1.17 (-1.42, -0.93) | 3.01×10^-19^ | -0.13 (-0.21, -0.06) | 8.06×10^-04^ |
| 5-Hydroxy tryptamine | -7.66 (-9.31, -6.03) | 3.01×10^-18^ | -1.01 (-1.54, -0.48) | 6.34×10^-04^ |
| PI(20:4/0:0) | 0.75 (0.59, 0.91) | 3.43×10^-18^ | 0.11 (0.06, 0.17) | 5.17×10^-05^ |
| Trp-Arg-Leu | -8.07 (-9.81, -6.32) | 6.99×10^-18^ | -0.37 (-0.93, 0.18) | 2.40×10^-01^ |
| ***Glycerophosphocholine** | -0.47 (-0.57, -0.37) | 1.46×10^-17^ | -0.04 (-0.08, -0.01) | 2.46×10^-02^ |
| 1,3-Octadiene | -0.37 (-0.45, -0.28) | 7.94×10^-16^ | -0.09 (-0.12, -0.06) | 2.84×10^-07^ |
| Undecan-3-ol | -16.35 (-20.33, -12.38) | 3.98×10^-14^ | -1.71 (-2.98, -0.43) | 1.59×10^-02^ |
| PG(15:0/14:0) | -0.68 (-0.84, -0.51) | 4.92×10^-14^ | -0.06 (-0.11, -0.01) | 4.03×10^-02^ |
| ***Lyso-PC(22:5)** | -1.19 (-1.48, -0.89) | 2.28×10^-13^ | -0.10 (-0.20, -0.01) | 7.09×10^-02^ |
| ***Lyso-PE(16:0)** | -0.43 (-0.53, -0.32) | 3.34×10^-13^ | -0.05 (-0.09, -0.02) | 8.29×10^-03^ |
| PE(20:0/0:0) | -0.28 (-0.35, -0.21) | 1.86×10^-12^ | -0.02 (-0.05, 0.01) | 1.40×10^-01^ |
| 2-Octenoylcarnitine | -7.67 (-9.67, -5.68) | 2.25×10^-12^ | -0.89 (-1.56, -0.22) | 1.59×10^-02^ |
| ***Lyso-PE(18:2)** | -0.46 (-0.58, -0.34) | 4.44×10^-12^ | 0.00 (-0.04, 0.04) | 9.84×10^-01^ |
| Lyso-PC(18:4) | -4.66 (-5.88, -3.43) | 5.24×10^-12^ | -0.07 (-0.47, 0.32) | 8.02×10^-01^ |
| ***Lyso-PC(10:0)** | -0.22 (-0.28, -0.16) | 9.09×10^-12^ | -0.01 (-0.02, 0.01) | 5.24×10^-01^ |
| ***Creatine** | 0.04 (0.03, 0.05) | 5.67×10^-11^ | 0.03 (-0.01, 0.07) | 1.77×10^-01^ |
| ***Lyso-PE(18:0)** | -1.17 (-1.49, -0.84) | 7.25×10^-11^ | -0.16 (-0.27, -0.05) | 1.00×10^-02^ |
| ***Isobutyrylcarnitine** | -2.49 (-3.19, -1.79) | 1.49×10^-10^ | 0.02 (-0.21, 0.25) | 8.93×10^-01^ |
| ***Lyso-PC(20:3)** | -0.19 (-0.24, -0.13) | 5.69×10^-10^ | -0.02 (-0.04, -0.01) | 4.08×10^-02^ |
| ***Tryptophan** | -0.02 (-0.03, -0.02) | 6.88×10^-10^ | -0.00 (-0.01, 0.00) | 1.05×10^-01^ |
| Lyso-PC(20:4) | -0.75 (-0.98, -0.52) | 4.88×10^-09^ | -0.08 (-0.16 -0.01) | 6.76×10^-02^ |
| ***Aspartic acid** | -0.14 (-0.18, -0.09) | 6.00×10^-09^ | -0.02 (-0.03, -0.01) | 1.81×10^-02^ |
| Acetylcarnitine | -0.34 (-0.45, -0.23) | 4.17×10^-08^ | -0.02 (-0.06, 0.01) | 3.06×10^-01^ |
| ***Valine** | -0.01 (-0.02, -0.00) | 5.53×10^-08^ | -0.03 (-0.04, -0.01) | 1.28×10^-02^ |
| Dicyclohexylamine | 23.99 (16.15, 31.84) | 7.51×10^-08^ | -0.21 (-2.87, 2.44) | 8.98×10^-01^ |
| ***Succinic acid** | -0.14 (-0.19, -0.10) | 1.22×10^-07^ | -0.03 (-0.04, -0.01) | 1.27×10^-03^ |
| ***Ethylchenodeoxycholic acid** | -0.32 (-0.43, -0.21) | 3.30×10^-07^ | -0.07 (-0.12, -0.04) | 3.17×10^-04^ |
| ***Propionylcarnitine** | -1.13 (-1.55, -0.72) | 2.71×10^-06^ | -0.01 (-0.15, 0.12) | 8.93×10^-01^ |
| Lyso-PE(22:5) | -2.75 (-3.77, -1.74) | 3.40×10^-06^ | -0.39 (-0.74, -0.04) | 4.43×10^-02^ |
| Lyso-PE(22:6) | -0.55 (-0.76, -0.35) | 4.35×10^-06^ | -0.11 (-0.18, -0.04) | 3.29×10^-03^ |
| PE(18:0/0:0) | -0.24 (-0.33, -0.14) | 2.43×10^-05^ | -0.02 (-0.05, 0.01) | 2.87×10^-01^ |
| Eicosatertraenoic acid | -4.15 (-5.84, -2.47) | 4.48×10^-05^ | 0.29 (-0.28, 0.87) | 3.68×10^-01^ |
| Lyso-PC(20:5) | -0.15 (-0.22, -0.09) | 5.57×10^-05^ | -0.04 (-0.06, -0.01) | 4.07×10^-03^ |
| ***Carnosine** | 0.07 (0.04, 0.11) | 1.13×10^-04^ | 0.07 (-0.04, 0.018) | 2.87×10^-01^ |
| ***Paraxanthine** | 0.12 (0.07, 0.17) | 2.19×10^-04^ | 0.03 (-0.02, 0.21) | 8.27×10^-01^ |
| 3-Methyl-2-buten×10-1-thiol | -11.12 (-16.45, -5.77) | 1.36×10^-03^ | -1.07 (-2.88, 0.73) | 2.94×10^-01^ |
| ***Carnitine** | 0.04 (0.02, 0.06) | 1.78×10^-03^ | 0.00 (-0.01, 0.01) | 9.39×10^-01^ |
| ***PG(13:0/14:0)** | -0.31 (-0.47, -0.15) | 3.54×10^-03^ | -0.01 (-0.06, 0.05) | 8.68×10^-01^ |
| M259T434 | 3.94 (1.85, 6.04) | 6.27×10^-03^ | 0.49 (-0.23, 1.19) | 2.34×10^-01^ |
| Methylglutarylcarnitine | 9.70 (4.30, 15.11) | 1.24×10^-02^ | -2.27 (-3.94, -0.61) | 1.47×10^-02^ |
| M727T404 | -9.74 (-15.46, -4.02) | 2.38×10^-02^ | -0.80 (-2.75, 1.14) | 4.82×10^-01^ |
| MG(18:0/0:0/0:0) | -0.23 (-0.36, -0.09) | 2.62×10^-02^ | -0.06 (-0.11, -0.02) | 1.37×10^-02^ |
| Lyso-PE(20:4) | -0.10 (-0.16, -0.04) | 2.68×10^-02^ | -0.03 (-0.05, -0.01) | 1.50×10^-02^ |
| PE(21:0/0:0) | -0.12 (-0.19, -0.05) | 3.41×10^-02^ | -0.02 (-0.05, 0.01) | 1.55×10^-01^ |

^a^ non-conditional logistic regression model discovered the association between metabolites and CVD risk

^b^ multiple linear regression model discovered the association between metabolites and HbA1c

*means that the metabolites were tentatively identified with reference compounds.

Table S2. HbA1c associated with incident cardiovascular disease mediated by first principal component in various metabolic pathway in young group.

| Metabolites | Pathway | PC1 (%) | Indirect effect (IE) | *P*_IE_ | Mediated proportion (%) |
| --- | --- | --- | --- | --- | --- |
| ***Fumaric acid** | Butanoate metabolism | 95.1 | 1.050 (1.019, 1.096) | 1.07×10^-2^ | 14.7 |
| ***γ-Aminobutyric acid** |  |  |  |  |  |
| ***Succinic acid** |  |  |  |  |  |
| ***Aspartic acid** | Alanine, aspartate and glutamate metabolism | 89.5 | 1.062 (1.018, 1.13) | 2.54×10^-2^ | 17.9 |
| ***γ-Aminobutyric acid** |  |  |  |  |  |
| ***Fumaric acid** |  |  |  |  |  |
| ***Succinic acid** |  |  |  |  |  |
| ***Fumaric acid** | Citrate cycle (TCA cycle) | 99.5 | 1.049 (1.019, 1.095) | 1.15×10^-2^ | 14.5 |
| ***Succinic acid** |  |  |  |  |  |
| ***Huppuric acid** | Phenylalanine metabolism | 89.7 | 1.075 (1.035, 1.133) | 2.64×10^-3^ | 20.6 |
| N-Phenylacetyl-L-glutamine |  |  |  |  |  |
| ***Fumaric acid** |  |  |  |  |  |
| ***Succinic acid** |  |  |  |  |  |
| Phosphatidylcholine | Glycerophospholipid metabolism | 94.7 | 3.561 (2.293, 5.853) | 8.26×10^-8^ | 86.5 |
| ***Lyso-PC(18:1)** |  |  |  |  |  |
| ***Phosphocholine** |  |  |  |  |  |
| ***Glycerophosphocholine** |  |  |  |  |  |

*means that the metabolites were tentatively identified with reference compounds.

Table S3. HbA1c associated with incident cardiovascular disease mediated by first principal component in various metabolic pathway in old group.

| Metabolites | Pathway | PC1 (%) | Indirect effect (IE) | *P*_IE_ | Mediated proportion (%) |
| --- | --- | --- | --- | --- | --- |
| ***Fumaric acid** | Butanoate metabolism | 94.9 | 1.040 (0.990, 1.108) | 1.92×10^-1^ | a |
| ***γ-Aminobutyric acid** |  |  |  |  |  |
| ***Succinic acid** |  |  |  |  |  |
| ***Aspartic acid** | Alanine, aspartate and glutamate metabolism | 90.6 | 1.129 (1.027, 1.26) | 2.33×10^-2^ | 29.2 |
| ***γ-Aminobutyric acid** |  |  |  |  |  |
| ***Fumaric acid** |  |  |  |  |  |
| ***Succinic acid** |  |  |  |  |  |
| ***Fumaric acid** | Citrate cycle (TCA cycle) | 99.4 | 1.039 (0.99, 1.107) | 1.97×10^-1^ | a |
| ***Succinic acid** |  |  |  |  |  |
| ***Huppuric acid** | Phenylalanine metabolism | 89.9 | 1.051 (0.99, 1.131) | 1.42×10^-1^ | a |
| N-Phenylacetyl-L-glutamine |  |  |  |  |  |
| ***Fumaric acid** |  |  |  |  |  |
| ***Succinic acid** |  |  |  |  |  |
| Phosphatidylcholine | Glycerophospholipid metabolism | 94.4 | 2.557 (1.418, 5.109) | 3.33×10^-3^ | 70.2 |
| ***Lyso-PC(18:1)** |  |  |  |  |  |
| ***Phosphocholine** |  |  |  |  |  |
| ***Glycerophosphocholine** |  |  |  |  |  |

a, Mediated proportion cannot be estimated due to the IE is not significant. *means that the metabolites were tentatively identified with reference compounds.

Figure S1


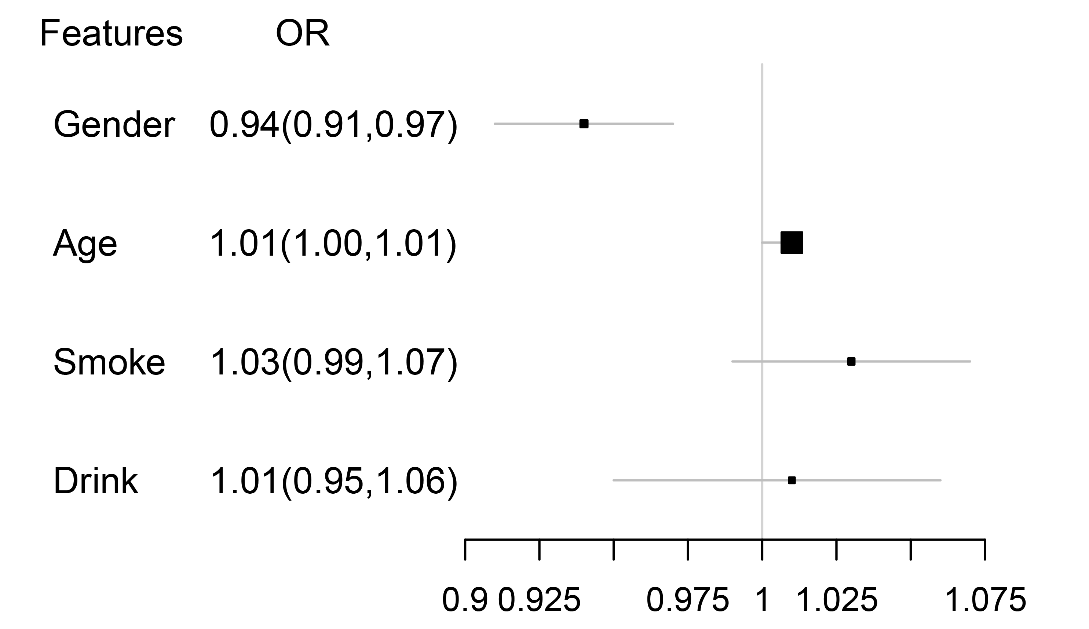


Figure S2


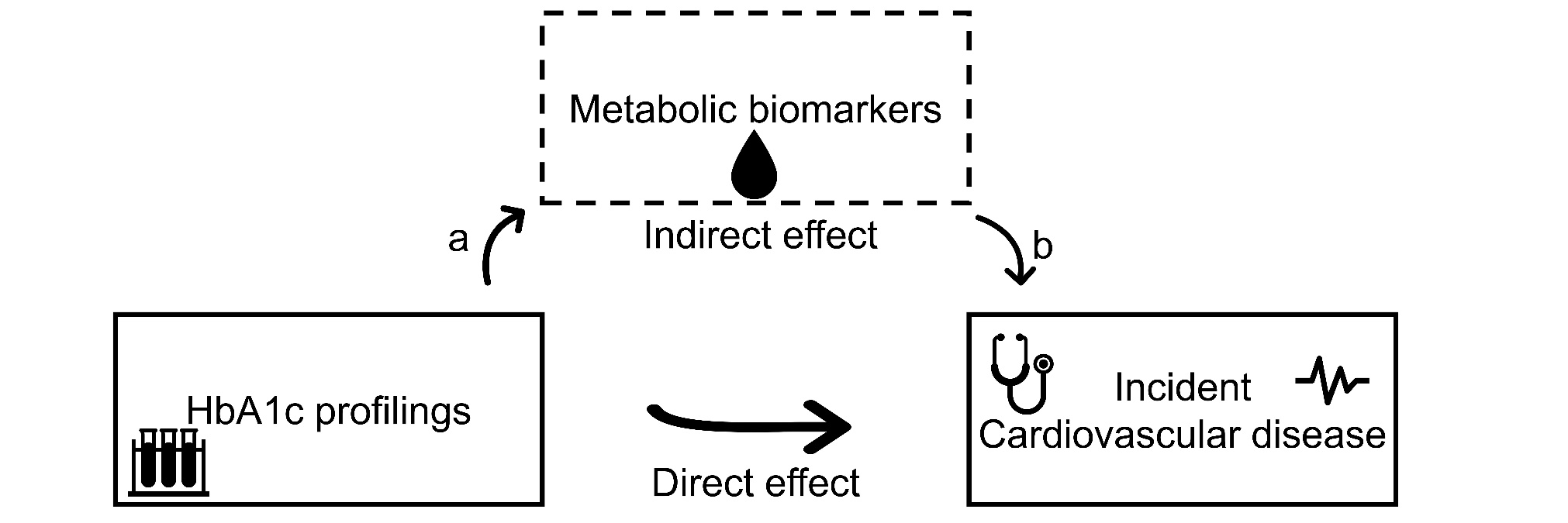

Supplement: Supplementary file 1 — Figure S1. Clinical characteristics. Figure S2. Schematic diagram of mediation analysis. Table S1. Metabolites associated with incident cardiovascular disease and HbA1c. Table S2. HbA1c associated with incident cardiovascular disease mediated by first principal component in various metabolic pathway in young group. Table S3. HbA1c associated with incident cardiovascular disease mediated by first principal component in various metabolic pathway in old group. [file CLC-42-934-s001.docx]
